# Blood Viscosity Changes in Diabetes Mellitus: A 20-Year Bibliometric Review and Future Directions

**DOI:** 10.7759/cureus.64211

**Published:** 2024-07-10

**Authors:** Jovita I Mbah, Phillip T Bwititi, Prajwal Gyawali, Ezekiel U Nwose

**Affiliations:** 1 School of Health and Medical Sciences, University of Southern Queensland, Toowoomba, AUS; 2 Dentistry and Medical Sciences, Charles Sturt University, New South Wales, AUS; 3 Public and Community Health, Amai Campus, Novena University, Ogume, NGA; 4 Health and Medical Sciences, University of Southern Queensland, Toowoomba, AUS

**Keywords:** whole blood viscosity, progress report, conceptual & empirical review, laboratory test, diabetes cardiovascular complications

## Abstract

Changes in hematological parameters due to diabetes are reflected in changes in whole blood viscosity (WBV). Understanding the impact of diabetes and its cardiovascular disease (CVD) complications can provide substantiation of how laboratory tests for WBV are useful to monitor the progression and treatment. The review examines research work done in the past 20 years to provide a framework for the present agenda.

This was a narrative review that followed the standard Scale for the Assessment of Narrative Review Articles (SANRA) approach. It includes both conceptual and empirical reviews. WBV was appraised in the context of bibliographic research on diabetes and other related factors such as metabolic syndrome (MetS) and oxidative stress.

The association of abnormal erythrocytes as well as the relationship between WBV and MetS is established. Changes in diabetes that contribute to the development of diabetic cardiovascular complications occur through the pathway of WBV physiology. However, longitudinal analysis is very limited.

There is a dearth of longitudinal study data on WBV in diabetes management. This lack of data justifies a need for further studies, especially prospective and retrospective analysis, to investigate the prevalence of diabetes mellitus about the prevalence of cardiovascular complications indices, especially estimated WBV (eWBV) between periods and within cohorts.

## Introduction and background

Research on WBV in diabetes: A 20-year progress review (2004-2024)

Given that the changes in hematological parameters due to diabetes are reflected in changes in whole blood viscosity (WBV), it is important to associate such changes with diabetic complications and management. Changes in several hematological parameters are related to the development of diabetic complications, for instance, increased differential cell counts, including neutrophils, eosinophils, and monocytes are indicators of the development of coronary artery disease and white blood cell (WBC) are suggested to play a role in the development and progression of diabetic complication [1]. In a study on the effects of red blood cell parameters on HbA1c and random blood sugar levels in diabetic diagnosis, there was a strong positive correlation between red blood cell parameters, HbA1c, and blood glucose that the authors concluded that red blood cell parameters are excellent tool parallel with HbA1c and blood glucose for assessment of diabetic cases [2]. This review will comprise two components including a brief narrative of the concepts and a critical empirical review of the blood viscosity phenomenon in diabetes.

There is a dearth of a longitudinal study of blood viscosity in the diabetes population, with perhaps the latest PubMed-archived report recommendation yet to be followed up i.e., blood viscosity as a hemorrheological indicator of CVD changes with glycemic control [3]. The discourse on blood viscosity dates back over 50 years ago when it was suggested that increased blood viscosity (hyperviscosity) is a major reason for reduced blood flow, which potentially leads to diabetic coma [4]. It has been established that ethnicity and geographical location may play a role [5,6]. At about the same time, Larcan et al. articulated that it is observable in diabetes mellitus (DM) due to various factors such as an increase in hematocrit and/or serum proteins, which may be associated with inflammation [7,8].

By 2003, diabetes research was established at Charles Sturt University, Australia, and “concentrated on assessing the degree of oxidative stress/antioxidants levels in people with disturbed blood glucose level (prediabetes) and type 2 diabetes subjects, compared to the normal controls” [9]. In 2004, the interest in cardiovascular complications [10-12], and the consideration of the WBV concept was integrated. Thus, it is now 20 years, hence worth reviewing what has been achieved to provide a clear framework for future direction.

Statement of the problem or the review objective

The Problem and Justification of Importance

There is a concern for evidence-based prescription of antiplatelet in diabetes [13]. The current diabetes management guidelines still recommend antiplatelet prophylaxis [14], at least for primary prevention of cardiovascular complications [15-17]. The recommendation includes the condition of contraindication [18], which is the risk of bleeding. While the International Normalized Ratio (INR) is used for anticoagulant therapy, the strategy needs the optimization of currently available methods, including laboratory methods such as WBV. Such strategy is of particular importance in rural and remote health services where affordances for INR are either lacking or limited, and estimated WBV (eWBV) can be obtained from routinely available full blood count and liver function tests at no further cost.

Objective of Review

The objective of this narrative review is to make a comprehensive summary of the milestone achievements from the 20 years of WBV research in diabetes. This is to establish a perspective of the progress made and what still needs to be done to advance the translation of this simple and universally available laboratory testing of eWBV for laboratory-based cardiovascular medicine monitoring in diabetes management.

## Review

Literature search methods

This was a focused traditional narrative literature review. The focus was on bibliographical publications in the blood viscosity research in diabetes at Charles Sturt University. Given cognizance of the systematic review being driven by hypothesis [19], which is not of interest, this review followed the Scale for the Assessment of Narrative Review Articles (SANRA) [20]. This is considered more appropriate to provide a comprehensive summary of research publications on the phenomenon of interest including the perspective on the scope of work done (conceptual and empirical). Nevertheless, an attempt was made to systematically search for literature on the PubMed platform, following the systematic approach [21], to identify relevant articles for appraisal that could add nuance to the summary of the progress review.

Results of the literature search

**Figure 1:**
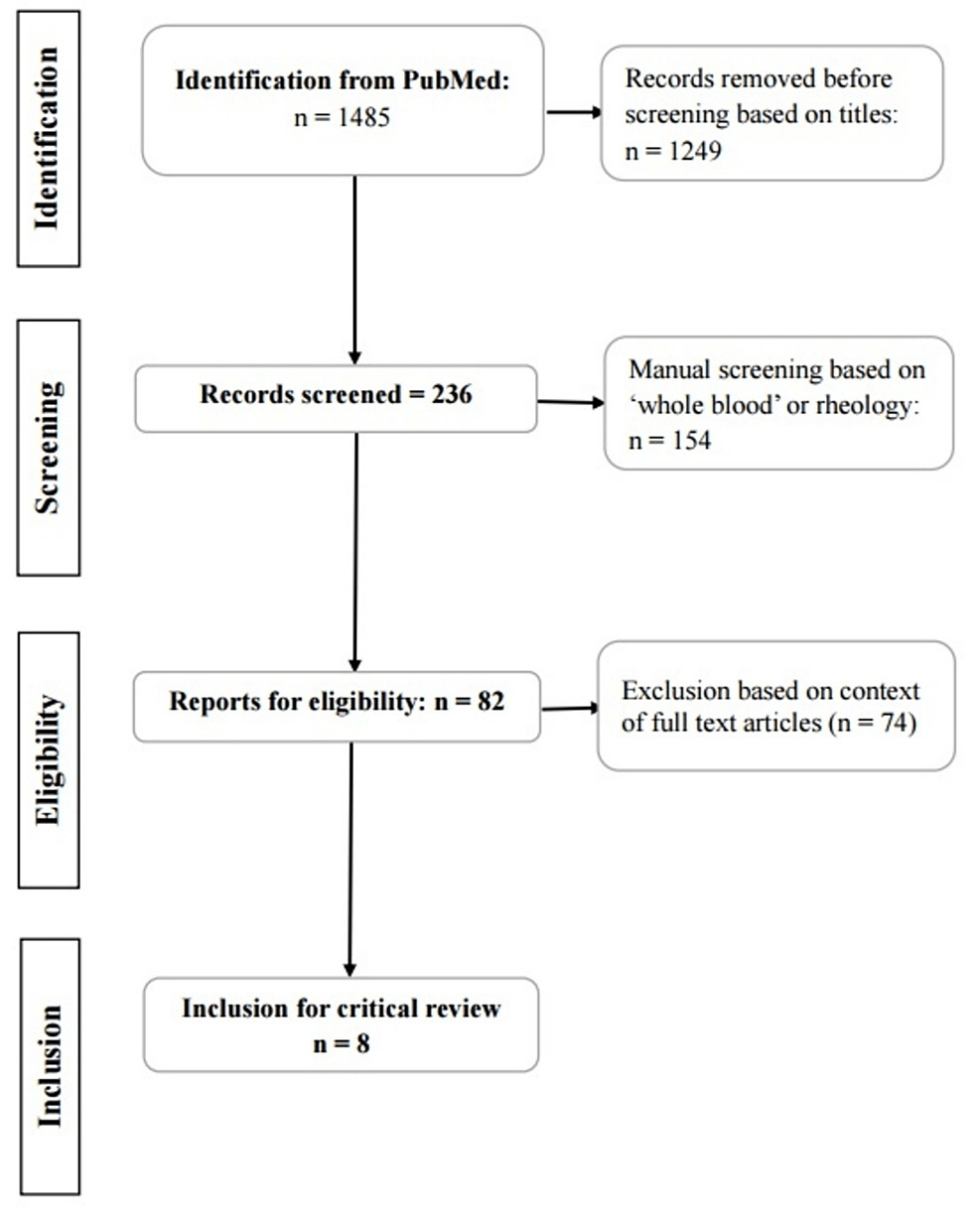
Dynamic flowchart summary of literature search on PubMed.

Figure 1 shows a summary of the literature search on PubMed. Table 1 provides the milestone research outcomes and scope. Among the 19 articles [22-40], six focused on diabetes were selected for appraisal and included in the eight articles on the Preferred Reporting Items for Systematic Reviews and Meta-Analyses (PRISMA) flowchart. Two additional pieces of literature indicated in Table 1 make up a total of eight on the flowchart. The eight articles selected for appraisal include three conceptual and five empirical.

**Table 1:** Milestone list of the bibliographical articles. ^‡^Selected for review. WBV, whole blood viscosity; eWBV, estimated whole blood viscosity; DM, diabetes mellitus

SN	Bibliography	2007	2008	2009	2010	2011	2012	2013	2014	2019	2022
1	DM impact: concept articulation^‡^ [22]	✓									
2	Oxidative stress indices (WBV) [23]		✓								
3	DM management: perspective in practice^‡^ [24]			✓							
4	WBV at different stages of DM^‡^ [25]				✓						
5	WBV prevalence in hyperglycemia^‡^ [26]				✓						
6	Other reports in the WBV series [27-33]				✓	✓					
7	WBV algorithm vs. viscometer methods [34]					✓					
8	Serum bilirubin and lipoproteinemia [35]						✓				
9	WBV in metabolic syndrome concept^‡^ [36]						✓				
10	Algorithm for risk assessment [37]							✓			
11	Vasculopathy triad [38]								✓		
12	WBV and HbA1c correlation^‡^ [39]									✓	
13	eWBV: sensitivity and specificity [40]										✓

Conceptual review

Diabetes-Induced Erythrocyte Oxidative Stress as a Cause of Increased Blood Viscosity

Many of the physiological processes that take place in our body produce harmful substances known as oxidants or free radicals, which are also beneficial in several ways. There are many radical species such as the reactive oxygen species (ROS) and the reactive nitrogen species in biological systems, of which the most widely studied is ROS. The body also produces antioxidants to neutralize the oxidant and maintain a balance. When the body cannot cope with the production of oxidative stress is an imbalance that occurs when the level of free radical production exceeds the capacity of the antioxidant defense, destruction of the cell membrane and erythrocyte lysis can lead to impaired actions of major enzymes, and block cellular processes necessary for normal function of the body [41], which includes impaired deformability leading to impairment in flow dynamics. It suffices that blood viscosity has been established as an intrinsic resistance of blood flow in the vascular system [42].

**Figure 2:**
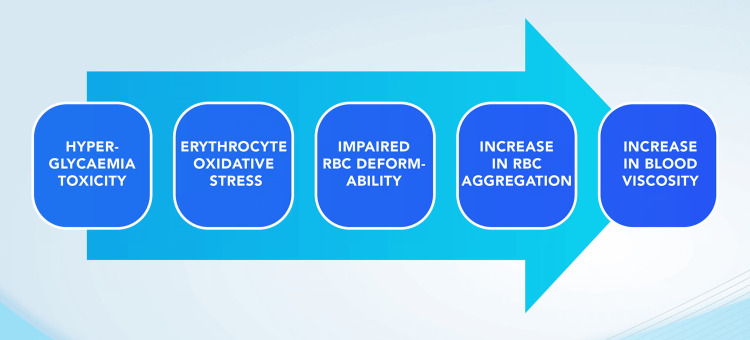
Concept of hyperglycemia as a cause of increased blood viscosity. Image credit: All authors.

The conceptual articulation to integrate blood viscosity study into diabetes research was premised on the knowledge that “pathophysiology of cardiovascular complications in diabetes involves hyperglycaemia-induced oxidative stress” and starts at the prediabetic stage. It is also presumed that “erythrocyte oxidative stress determination may provide an additional marker for both preclinical and advanced disease” in diabetes pathogenesis. Thus, the graphical flowchart vis-à-vis the concept of hyperglycemia (re: DM) being a potential cause of hyperviscosity was developed (Figure 2). It describes how erythrocyte oxidative stress, which arises from DM, can cause cardiovascular complications, including increased blood viscosity [22].

WBV and metabolic syndrome

Metabolic syndrome (MetS) is related to DM and is characterized by association with dyslipidemia, hypertension, and obesity among others [43]. It is the clustering of cardiovascular risk factors such as dyslipidemia, hypertension, abdominal obesity, and insulin resistance [44].

**Figure 3:**
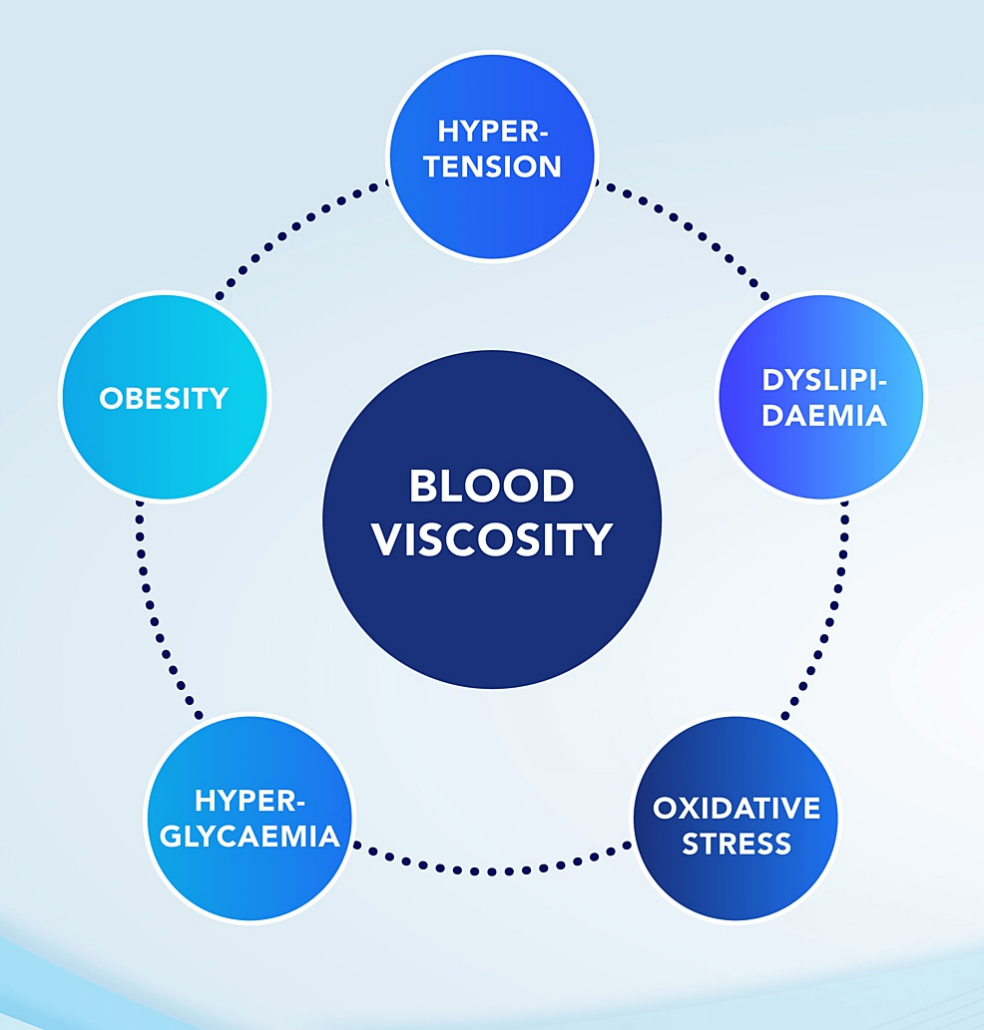
Concept of oxidative stress and metabolic syndrome in blood viscosity. Image credit: All authors.

In this conceptual review [36], which also forms the basis of WBV evaluation in stroke [45], a critical look at the effect of WBV on different components of MetS was evaluated and substantiated (Figure 3). For instance, calculated WBV was found to be positively correlated with fasting plasma glucose and serum insulin level. Even after adjustment for age, sex, and parental history of DM, type 2 DM remains strongly associated with WBV. This study also suggests that WBV is associated with insulin resistance and is an independent predictor of type 2 DM.

Literature evidence of microcirculation deterioration in diabetes

Erythrocyte deformability is of extreme importance in microcirculation. As the lumen of small blood vessels is in the region of 4-6 µm and the erythrocyte is approximately 8 µm, deformability is crucial for the erythrocyte to pass through [46].

**Figure 4:**
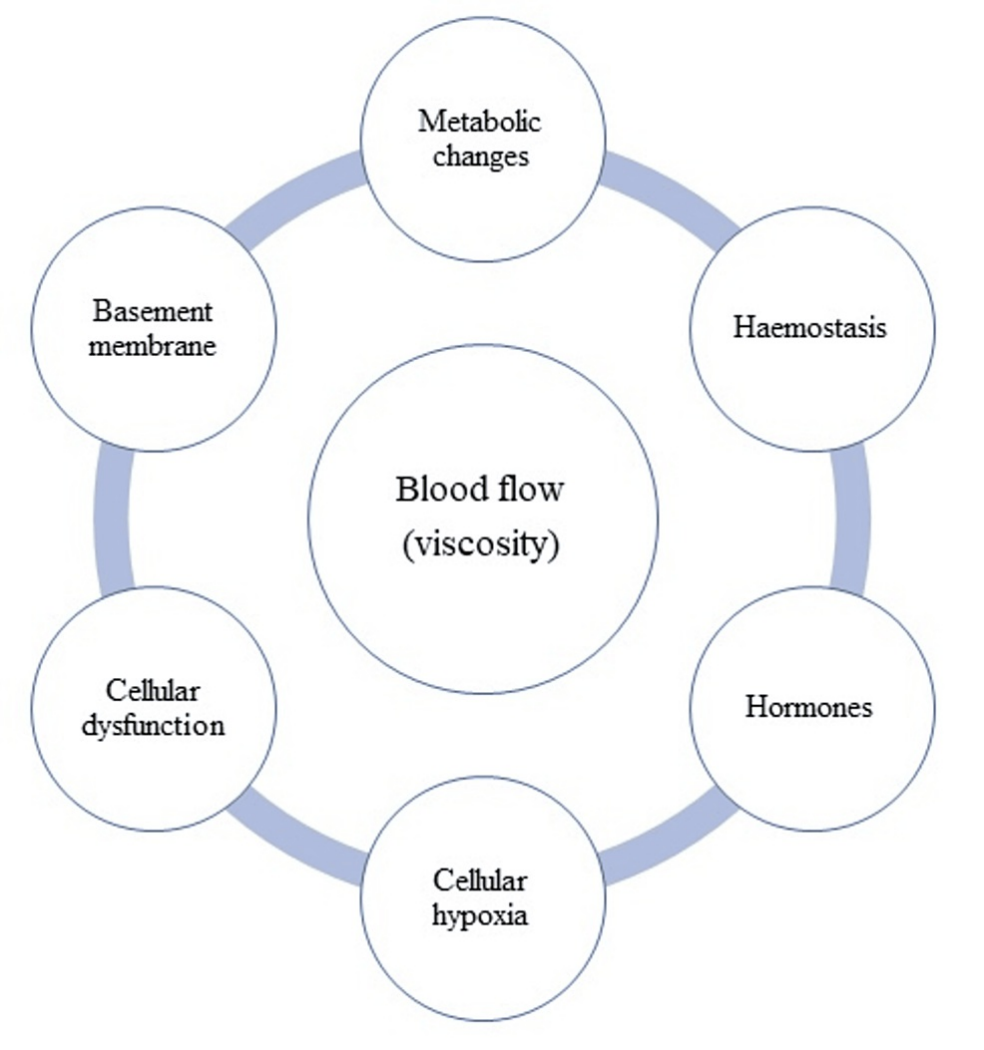
Concept of the factors impacting microcirculation. Image credit: All authors.

In this conceptual review depicted in Figure 4, the author re-examined the widespread progressive deterioration of the entire microcirculation in the context of the contributions from seven aspects of diabetic changes, namely, alterations in the basement membrane, cellular function, cell metabolic changes, blood flow properties, disturbed homeostasis, oxygen transport, and hormone production [47].

Empirical reviews

WBV Determination in Diabetes Management: Perspective and Practice

There are evidence-based studies that support the efficacy and cost-effective benefits of nutrition therapy as a component of quality diabetes care [48]. Nutrition counseling can work to improve and maintain glycemic targets, reducing cardiovascular risk.

**Figure 5:**
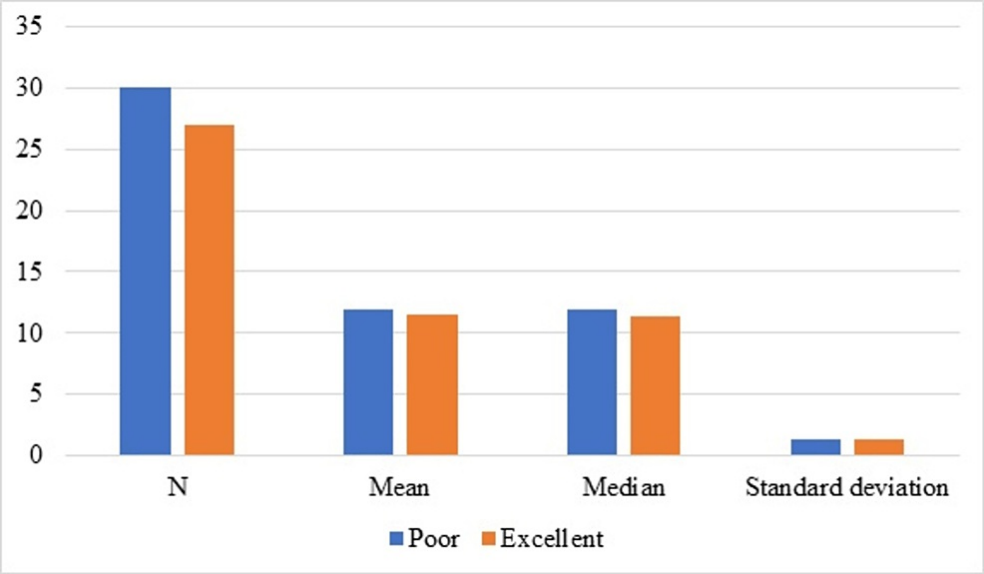
Whole blood viscosity of poor and excellent glycemic control. Image credit: All authors.

In the retrospective study, the agenda of the investigation was whether the two guidelines that recommended the identification of deficiency in antioxidant vitamins and condition of no contraindication for nutritional and antiplatelets were observed in clinical settings [24]. There has been speculation of seasonal variations [49], which was not investigated. The study evaluated 57 cases [24], of which 30 were from poor glycemic control and 27 from excellent glycemic control, selected based on serum total protein and hematocrit results, and WBV levels were calculated (Figure 5).

The study results showed that neither of the two guidelines was observed, as none of the 57 cases (from poor/excellent glycemic control groups) requested any antioxidant vitamins or WBV. WBV was significantly lower in the group with excellent glycemic control. Antiplatelet and antioxidant nutritional therapies were found to be alternatives to aspirin, impacting WBV.

WBV prevalence in hyperglycemia

Increased blood viscosity reduces blood flow and raises vascular resistance in the cardiovascular system. This, in turn, leads to systemic hypertension and ischemic organ damage [50]. As hematocrit is the most important factor in blood viscosity, blood is most likely to be more viscous in the vascular beds of the kidney than in any other organs [51]. This means that the harmful effects of elevated blood viscosity are expected to manifest particularly in the kidneys.

In this review, the authors investigated the prevalence of WBV in hypercreatinemia, hyperglycemia, and hyperlipidemia. The purpose was to determine the proportion of patients who may not require antiplatelet therapy [26]. Using relatively large archived clinical pathology data that included tests on blood glucose, creatinine, lipid profile, hematocrit, and total protein, the report indicated that eWBV levels were statistically significant in all abnormal biochemistry values compared to normal biochemistry values (Table 2).

**Table 2:** BGL in normoglycemia and distribution of eWBV. BGL, blood glucose level; eWBV, estimated whole blood viscosity

	Year of data	Diabetic BGL	Prediabetic BGL	Normal BGL
Normo-viscosity in stratified BGL groups	YR2000	91.0%	94.6%	95.7%
	YR2002	94.7%	95.2%	95.3%
	YR2004	95.1%	96.0%	96.3%
	YR2006	94.2%	93.7%	94.5%
	YR2008	95.3%	95.7%	96.1%
	Average*	94.1%	95.0%	95.6%
	Year of data	eWBV_high	eWBV_low	eWBV_normal
Distribution of eWBV categories in diabetic BGL	YR2000	7.2%	1.8%	91.0%
	YR2002	2.0%	3.3%	94.7%
	YR2004	3.7%	1.2%	95.1%
	YR2006	3.5%	2.3%	94.2%
	YR2008	2.5%	2.2%	95.3%
	Average	3.8%	2.2%	94.1%

The findings showed that hyperviscosity in people with evidence of chronic disease was low, although this low prevalence was statistically significantly different compared to people with normal biochemistry results. A critical review of data regarding diabetes showed observations in Tables 1 and 2. Analysis showed that higher WBV was statistically significantly associated with hyperglycemia, observed only between the first and fourth quartiles. The implication is that the current disregard for WBV may be due to its perceived low sensitivity, which ignores its higher specificity [37,40], as well as the importance of monitoring hyperviscosity syndrome for its validity and reliability in assessing bleeding risk [52].

WBV at different stages of diabetes progression

In another study, the levels of WBV at various stages of diabetes were compared [25]. The study was premised on the proposition that while oxidative stress due to hyperglycemia albeit DM can be a cause of hyperviscosity diabetic cardiovascular complications, it was unknown *whether or not there is variation in WBV at different stages of diabetes mellitus*. Oxidative stress and WBV levels were compared between participants at different stages of diabetes status (apparently healthy, prediabetes, diabetes, and diabetic-CVD groups).

**Figure 6:**
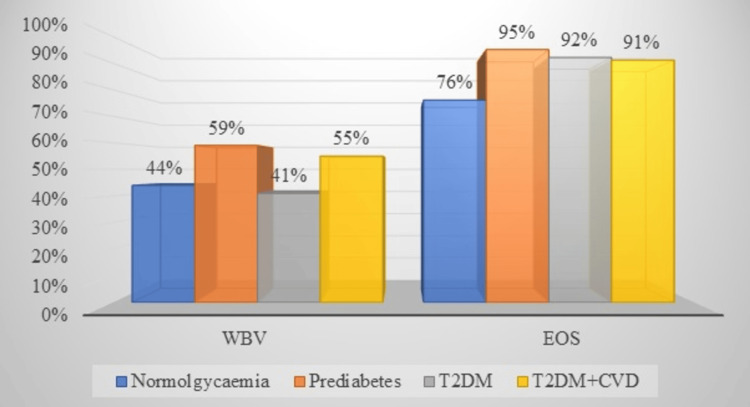
WBV levels at different groups and association with oxidative stress. Image credit: All authors. T2DM, type 2 diabetes mellitus; CVD, cardiovascular disease; WBV, whole blood viscosity

The results showed that WBV was significantly higher in prediabetes compared to the control group (*P *< 0.01) and DM+CVD group (*P *< 0.04). Interestingly, the DM+CVD group did not significantly differ from the prediabetes groups. Further, there was about 76% prevalence of oxidative stress associated with high WBV in the general cross-sectional study population, and 95% prevalence in the prediabetes group. The study showed that WBV varies between individuals with different stages of diabetic macrovascular pathogenesis, including prediabetes (Figure 6).

Hemorheological disorders in DM

Whole blood behaves as non-Newtonian fluid as a result of a large quantity of cells, mostly red blood cells, suspended in the plasma. Viscosity is dependent on the shear rate and is greatly affected by hyperglycemia seen in diabetes. Anything that affects viscosity will, therefore, affect the hemorheological properties of blood.

In this empirical review [53], the study examined key hemorheological parameters such as WBV, erythrocyte deformability, and aggregation about elevated glucose levels seen in diabetes. It also explored how this increased viscosity affects microcirculation. The authors presented a list of 37 empirical reports, which state that WBV is statistically significantly lower in apparently healthy individuals compared to groups of people living with diabetes (Table 3).

**Table 3:** Descriptive statistics of WBV levels for control and diabetes mellitus groups. WBV, whole blood viscosity

	Control	Diabetics
Mean	7.59	8.98
Standard error	0.95	1.15
Median	5.88	6.38
Standard deviation	5.64	6.82
Sample variance	31.77	46.55
Kurtosis	8.62	7.13
Skewness	2.62	2.41
Range	29.03	33.85
Minimum	2.27	2.55
Maximum	31.3	36.4
Sum	265.72	314.18
Count	35	35

Blood viscosity changes with HbA1c

In one of the most recent empirical *proof-of-concept *evaluations, the association between WBV and HbA1c was investigated. The aim was to determine the correlation of age with HbA1c as well as age and WBV in patients with diabetes. The study compared HbA1c and WBV between age groups, as well as their correlations with age, and the odds that the variables would increase with age. The results showed that aging correlated negatively with HbA1c (*r* = -0.25), but positively with WBV among the oldest age group (*r* = 0.27). Anemia was noted to appear as a confounding on the odds ratio. Further, HbA1c and WBV appeared to be positively correlated but only in patients with good HbA1c levels (*r* = 0.39). The study concluded with a suggestion to rethink the notion that both HbA1c and WBV increase with age among individuals living with diabetes [39].

Discussion

With the first conceptual review [22], further studies articulated that subclinical cardiovascular disease (SCVD) precedes symptomatic complications in diabetes and is associated with oxidative damage. Hence, assessment and management of oxidative damage was deemed to be imperative. Nevertheless, in the study that investigated “biomarkers associated with CVD, diabetes and oxidative stress to determine a set of indices that could be useful to assess oxidative damage in diabetic macrovascular pathogenesis” [23], D-dimer but not WBV was evaluated. Therefore, this constituted one of the limitations at the beginning of the WBV research in diabetes.

With regards to MetS, it can be inferred that obesity, hypertension, and DM produce a state of chronic inflammation and oxidative stress. This condition leads to a decrease in erythrocyte deformability and morphological abnormality of erythrocyte which ultimately leads to elevated WBV. Raised WBV will cause an increase in the pump requirement of the heart, resulting in an increased risk of CVD. There is sufficient evidence that components of oxidative stress cause a decrease in erythrocyte deformability and an increase in WBV, leading to an increased CVD risk [54]. However, the precise changes in WBV levels among individuals undergoing diabetes management have to be elaborately investigated.

The deterioration of microcirculation in diabetes does not seem to be caused by one type of change or alteration but by an interaction of one or more categories of changes. The fact that deterioration progresses over a long period shows that a mixture of adaptation and degeneration occurs in other to respond to several burdens placed on microcirculation by diabetes. In determining which alterations or changes are more important in managing the burden, priority should be given to those changes that are pathogenic and produced by the diabetic state, rather than those that develop after complications, such as microangiopathy, have appeared. Treatment at this stage should be restricted to control of hyperglycemia to prevent the development of microangiopathy. Prevention can only be thought of after understanding the process by which microvascular damage develops.

The finding is failure to follow the two guidelines in clinical practice, and this was attributed to a lack of awareness on the part of the clinicians that such tests are not only available but easily accessible at no additional cost to the patients. Second, the controversy surrounding the efficacy of antioxidant nutritional therapy has not helped matters. The conclusion is that the important consideration of managing oxidative stress is being overlooked [24].

Cognizance must be taken of potential seasonal variation [49]. That is, on one hand, utilizing available laboratory tests for assessing antioxidant vitamin status in patients still constitutes a gap in knowledge and practice. On the other hand, the possibility of seasonal differences in reference values requires further investigation.

The population exhibits 97.5% within normal WBV levels. However, Table 1 shows that the diabetic group has the lowest proportion (94.1%) of normal eWBV, while the normoglycemic group has the highest proportion (95.6%) of normo-viscosity, although statistical significance is not achieved (*P* = 0.178). Table 2 shows that among the population with diabetic blood glucose level (BGL), 3.8% have hyperviscosity, which implies prothrombotic risk, while 2.2% have hypoviscosity and are at risk of bleeding. The potential difference in seasonal reference values constitutes a research hypothesis to investigate.

From this bibliographic review, it was identified that WBV in MetS has been investigated, which provided the basis for the second conceptual framework (Figure 3). Pertinent to the concept is that abnormal erythrocyte morphology increases in conditions of oxidative stress and chronic inflammation, leading to a decrease in normal biconcave cells, as seen with acanthocytes, echinocytes, and stomatocytes. These abnormalities, especially erythrocyte physiology leading to increased WBV, could be because of the effect of free radicals on the erythrocytes [36]. Understanding the changes in erythrocyte morphology and deformability is crucial to blood flow and the risk to the cardiovascular system. Oxidative stress and inflammation increase the number of abnormal erythrocytes possibly due to the effect of free radicals generated. This abnormal erythrocyte can pose a problem in the circulation.

It is established that in diabetes "erythrocyte deformability is reduced, whereas its aggregation increases, both of which make whole blood more viscous compared to healthy individuals" [53]. At higher shear rates, WBV was generally higher for the diabetic group compared to the control. In addition, hematocrit was higher in the diabetic group. The altered blood flow might encourage stagnation of blood flow in the capillaries and post-capillary venules in diabetic patients. Elevated viscosity and reduced blood flow in small blood vessels can lead to vital organs being deprived of oxygen, resulting in hypoxia, acidosis, and microvascular damage. Therefore, DM is characterized by elevated WBV. Hyperglycemia from diabetes affects the modification of erythrocyte morphology and internal structure, leading to reduced blood flow in the microcirculation, which, in turn, contributes to tissue and organ damage.

Last but not least, the report on HbA1c correlation with eWBV suggests two implications: a confounding factor and a reevaluation of existing notions. These suggestions imply that further studies are warranted. Given the availability of longitudinal data [55], this calls for additional evaluation of the records.

## Conclusions

DM is characterized by elevated blood glucose levels, and this review examined the impact of this raised blood glucose from a hemorheological standpoint. High blood glucose leads to oxidative stress, which, in turn, leads to alteration of erythrocyte morphology, deformability, aggregation, and function. This can result in elevated WBV and reduced blood flow, especially in microcirculation, posing risks to the cardiovascular system. The monitoring of WBV can aid in early diagnosis and management of cardiovascular complications of diabetes care, especially considering that individuals living with diabetes are commonly on antiplatelet medications for prevention.
